# Remote Sensing Low Signal-to-Noise-Ratio Target Detection Enhancement

**DOI:** 10.3390/s23063314

**Published:** 2023-03-21

**Authors:** Tian J. Ma, Robert J. Anderson

**Affiliations:** Sandia National Laboratories, Albuquerque, NM 87185, USA

**Keywords:** remote sensing, remote sensing detection, target detection, low SNR target detection, low signal-to-noise ratio target detection, low SNR target tracking, low signal-to-noise-ratio target tracking, multi-frame moving object detection system, MMODS

## Abstract

In real-time remote sensing application, frames of data are continuously flowing into the processing system. The capability of detecting objects of interest and tracking them as they move is crucial to many critical surveillance and monitoring missions. Detecting small objects using remote sensors is an ongoing, challenging problem. Since object(s) are located far away from the sensor, the target’s Signal-to-Noise-Ratio (SNR) is low. The Limit of Detection (LOD) for remote sensors is bounded by what is observable on each image frame. In this paper, we present a new method, a “Multi-frame Moving Object Detection System (MMODS)”, to detect small, low SNR objects that are beyond what a human can observe in a single video frame. This is demonstrated by using simulated data where our technology-detected objects are as small as one pixel with a targeted SNR, close to 1:1. We also demonstrate a similar improvement using live data collected with a remote camera. The MMODS technology fills a major technology gap in remote sensing surveillance applications for small target detection. Our method does not require prior knowledge about the environment, pre-labeled targets, or training data to effectively detect and track slow- and fast-moving targets, regardless of the size or the distance.

## 1. Introduction

The volume and velocity of Big Remote Sensing Data poses a significant computational and storage challenge to modern applications [1]. If data are not transformed into immediate information, the generating of actionable intelligence will be delayed. This challenges the human capacity to store and review the data after the fact [2]. Detecting small objects in real time using remote sensors is an ongoing and challenging problem [3]. One challenge is where an object(s) is located far away from the sensor, in which its size naturally appears to be much smaller. A sensor’s sensitivity diminishes as the distance from the target increases. Another key challenge is when the environmental conditions can be dynamic (i.e., weather conditions, sunlight, obstructions, etc.). Poor environmental conditions (e.g., low visibility) can reduce the visual quality of a target(s). A combination of these factors can contribute to the target having a low Signal-to-Noise Ratio (SNR). There is also the challenge of the Limit of Detection (LOD), whereby the remote sensors are bounded by what is observable in each image frame. In other words, what a human sees in a single image frame is limited by what the sensor captures in one frame. When a small foreground object is very close to the background noise (e.g., low SNR), humans cannot accurately observe and label the data. Therefore, it cannot be used to train a machine classifier, where it helps the machine learn to identify similar objects in the future. In this paper, we present a new method, a “Multi-frame Moving Object Detection System (MMODS)”, to overcome the LOD of modern remote sensing in a detection system. 

Deep learning methods have gained wide popularity in object recognition [4]. For example, methods such as You Only Look Once (YOLO) [5] and Mask R-CNN [6] have shown that they can achieve high accuracy for “large size” object identification. Small size object identification remains a significant challenge [7]. Machine learning methods are generally applicable to targets containing high SNR and high resolution that relies on human-observed labeled data. When the target’s SNR of a remote sensor is close to the noise and the object size is too small (few pixels), humans cannot accurately observe and label the data for a machine classifier to be trained. The lack of features on a target makes it undesirable to model the machine detector using a deep neural network with a high risk of overfitting on the noise.

Traditional real-time moving object detection techniques typically involve “Background Subtraction” [8]. As image frames are continuously flowing into the system, an estimate of the background is computed at each time step. When a new frame arrives, the background estimate from the previous frame will be subtracted to produce a “Difference Frame”. Thresholding can then be applied on the Difference Frame to provide foreground and background discrimination [9]. Popular background estimation approaches such as the Gaussian Mixture Model (GMM) [10] attempts to estimate an image pixel’s background intensity using multiple Gaussian models. Principal Components of Pursuit (PCP) [11] attempts to decompose an image into sparse and low-rank components, where the low-rank components correspond to the stationary background. Subspace tracking techniques [12] model the background as a linear combination of basis images, where the background’s basis vectors are updated at each time step with a forgetting factor. Despite many recent advances in change detection [13], the frame-by-frame change detection approach is insufficient to detect low SNR targets with a manageable false alarm rate. By dropping the detection threshold close to the noise level, it will result in high levels of false alarms. Thus, there is a need to enhance the target’s SNR before applying a detection threshold.

Prior work has been carried out to improve the target’s SNR through matching and the integration of target signals. Reed et al. [14] introduced a 3D matched filter method to enhance the SNR of a constant velocity moving target by integrating the target’s signal according to its constant velocity motion over a framed sequence. This method assumes a known number of targets and its constant motion trajectory. The Track-Before-Detect (TBD) approach [15] incorporates the use of dynamic motion modeling, such as the Kalman Filter [16,17] and the Particle filter [18,19], to predict and integrate target motion over multiple frames to enhance a target’s SNR. However, the TBD approach is generally limited to slow moving targets as performance tends to degrade for high-speed targets [20]. A TBD approach using multiple motion models [21] has been demonstrated in simulations to better track maneuverable targets. However, it is not clear how the technique performs against real data. More recently, a constrained velocity filter approach [3] demonstrated significant improvement in low SNR target detection using real data collected by a remote video camera. This method uses a combination of known path constraints and the target’s motion model to improve its SNR by integrating target signals over a pre-determined path. However, the requirement to have a pre-determined path hinders the ability to apply the method in unconstrained areas or when a path constraint is unknown. 

In this paper, we introduce a new multi-frame detection to enhance a target’s SNR. Our method demonstrates a significant improvement over the traditional single-frame detection approach. 

## 2. Materials and Methods

### 2.1. Materials

**Figure 1:**
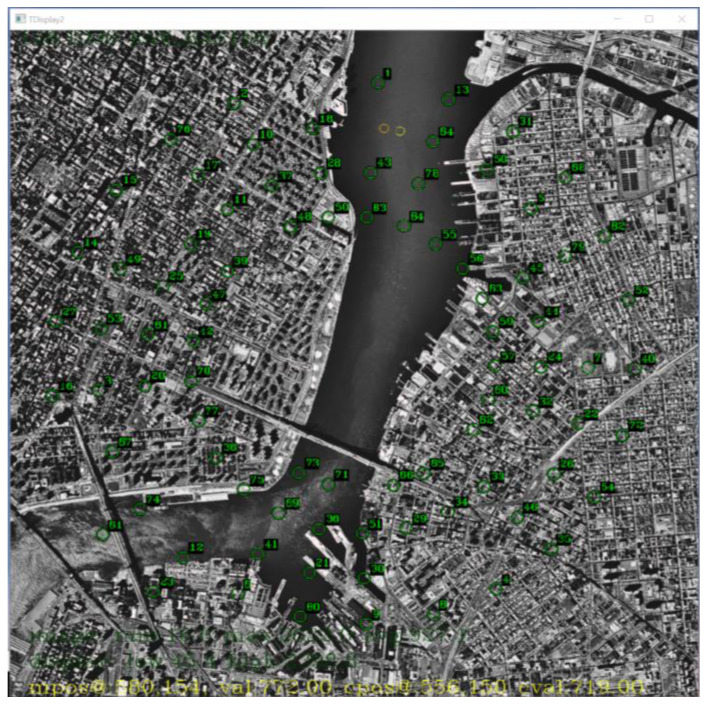
Simulated Raw Image Frame with 100 targets traveling in a circular motion.

To measure the performance of our algorithm, we simulated a scenario where 100 targets with low SNR were traveling in a circular motion at various speeds and accelerations. The targets were uniformly distributed between pixel intensities of 10 to 50. The point target injected was a Gaussian curve with a radii uniformly distributed between 1.0 and 1.6. Gaussian noise was added across the base image with a mean intensity of 10. The experiment setup resulted in targets with SNRs between 1 and 5. A summary of the experiment setup is depicted in Table 1 and the simulated frame is depicted in Figure 1. Injected target positions are indicated by a green circle in Figure 1. 

**Figure 2:**
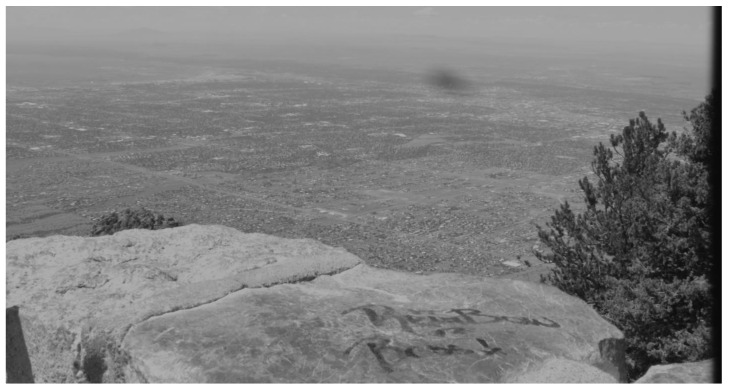
Video Frame captured from the Peak of Sandia Mountain.

We also collected data using a video camera that was mounted at the peak of Sandia Mountain to view traffic on the ground. The vehicles on the road(s) were so small that it was not visible to the human eye in the video frame shown in Figure 2. The camera location and specifications are summarized in Table 2 and Table 3. 

### 2.2. Method

We invented a new detection approach that combines multi-frame object detection processing with a dynamic target motion estimation algorithm. Our method enhances a target’s SNR by finding, matching, and integrating target signals over a temporal framed sequence. SNR enhancement is made possible because moving target signals are correlated over a temporal framed sequence, but the noise is generally uncorrelated (e.g., random). Hence, when correlated signals are integrated, they increase by a linear factor. However, the integration of random noises does not increase by the same amount. 

**Figure 3:**
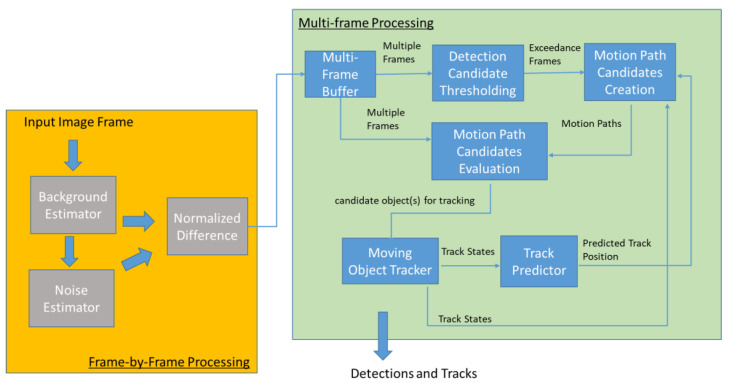
Multi-frame Moving Object Detection System.

The workflow of our Multi-frame Moving Object Detection System (MMODS) is depicted in Figure 3. Arrows in the figure is used to show data flow between components. Processing components that are built by leveraging existing scientific work are denoted in gray. Components resulting from our scientific contribution processing are denoted in blue. 

#### 2.2.1. Frame-by-Frame Processing

As the frames are continuously flowing into the system, we estimate the background using a simple Infinite Impulse Response (IIR) lowpass filter.

(1) $$B\left(t\right)=\left(1-\alpha \right)B\left(t-1\right)+\alpha F\left(t\right)$$

where $B\left(t\right)$ corresponds to the background frame computed at time t, $F\left(t\right)$ corresponds to the frame at time t, and α corresponds to the update rate [0,1].

The difference image for each time step can be simply computed by subtracting the current frame with the estimated background from the previous time step.

(2) $$D\left(t\right)=F\left(t\right)-B\left(t-1\right)$$

where $D\left(t\right)$ corresponds to the Difference Frame at time t, $F\left(t\right)$ corresponds to the frame at time t, and $B\left(t-1\right)$ corresponds to the background computed in the previous time step. 

To model the estimated background deviation, we estimate the temporal variance v  of frame at each time step t using a similar IIR low pass filter:

(3) $$v\left(t\right)=\left(1-\gamma \right)\;D{\left(t\right)}^{2}+\gamma \;v\left(t-1\right)$$

where γ is the variance update rate of [0,1]. The temporal standard deviation σ for pixel (i,j) at time t is obtained using the following equation:

(4) $$\sigma \left(i,j,t\right)=\sqrt{v\left(i,j,t\right)}$$

Difference Frame normalization [3] is applied to ensure pixel intensity across all image regions are normalized with respect to the estimated temporal noise. This is a pre-requisite for signal integration methods, as shown in [3]. Using similar notations in [3], Normalized Difference Frame $N_{d}$ for framed pixel location $\left(i,j\right)$ in time t is expressed as follows:

(5) $$N_{d}=\frac{D\left(i,j,t\right)}{\sigma \left(i,j,t-1\right)}$$

where $\sigma \left(i,j,t-1\right)$ represents the temporal standard deviation for the framed pixel location $\left(i,j\right)$ at the previous time step.

#### 2.2.2. Multi-Frame Processing

##### Frame Buffer

We created a temporal memory buffer to store a running temporal window of *Normalized Difference* frames so that multi-frame processing could be utilized in later steps.

##### Detection Candidate Thresholding

**Figure 4:**
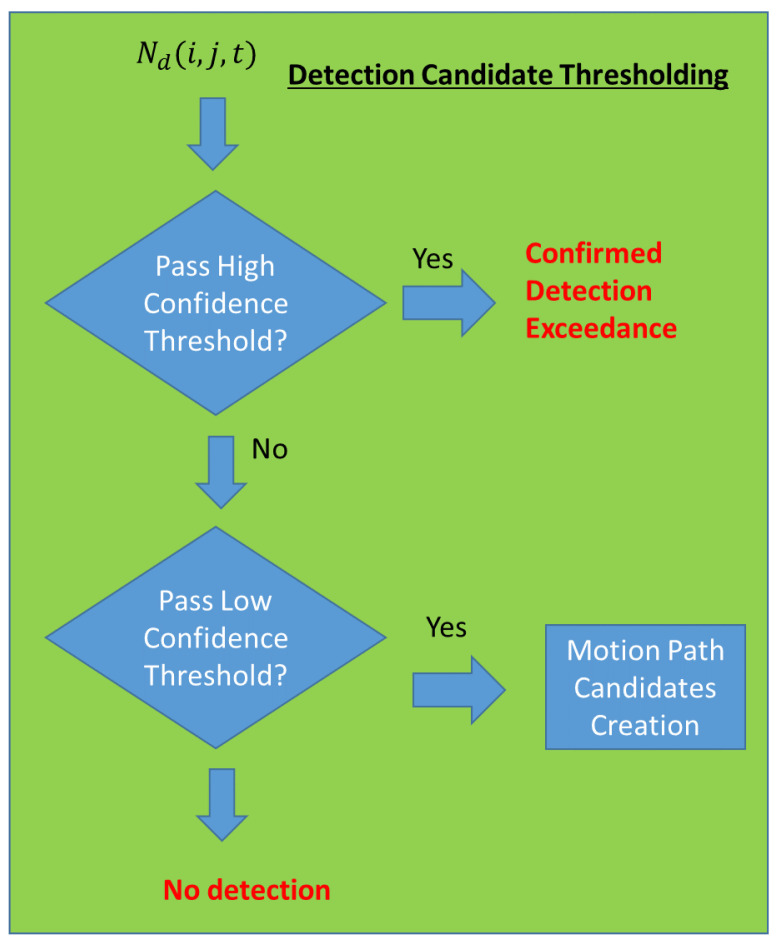
Detection Candidate Thresholding.

Thresholding is applied on the Normalized Difference Frame by comparing each pixel values in the Normalized Difference Frame with a “high” threshold. If the pixel value in the Normalized Difference Frame exceeds the “high” threshold, SNR enhancement is not needed because this is already a high SNR target. However, if the pixel value falls between the “low” threshold and the “high” threshold, the detected candidate pixels will undergo signal enhancement to increase the target’s SNR. The logical flow for this component is depicted in Figure 4. 

##### Motion Path Candidates Creation

**Figure 5:**
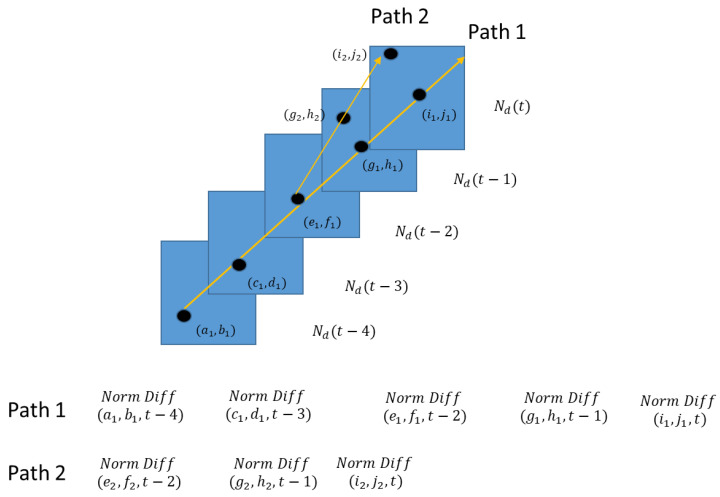
Motion Path Creation Illustration. Note, in this example, our approach adds a delay to the overall processing by two frames.

Motion paths are created by using detection exceedance from prior frames and post frames. Having an equal number of prior and post frames is necessary, so that the signal enhancement is not overweighted towards the past or the future. This is accomplished by casting probable motion paths between exceedances over the framed sequence. For example, suppose we use a frame buffer size of five frames. At time t, we have the following Normalized Difference Frames in the running buffer, $N_{d}\left(t-4\right)$, $N_{d}\left(t-3\right)$, $N_{d}\left(t-2\right)$, $N_{d}\left(t-1\right)$, and $N_{d}\left(t\right)$. To find motion paths at time t−2, we form possible motion paths using detection candidates from prior frames t−4, t−3, and post frames t−1, t. An illustration of using detection candidates to create motion paths is depicted in Figure 5.

Once object tracking starts, motion paths can also be created using the predicted state vector from the Moving Object Tracker; see Figure 3. For our approach, a Kalman Filter is used for object tracking [22]. The dynamic of the object is modelled as such:

$$x=\left[\begin{array}{c} r\\ \dot{r}\\ \ddot{r}\\ c\\ \dot{c}\\ \ddot{c} \end{array}\right]$$

where $r,\dot{r,}\ddot{r},\;c,\;\dot{c},\;\ddot{c}$, represent the position, velocity, and acceleration state vectors in row and column directions. 

**Figure 6:**
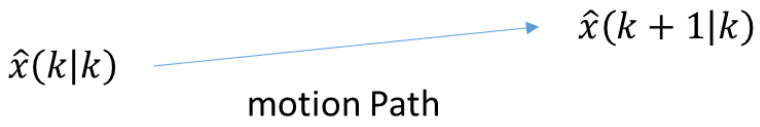
Motion Path Creation Using Tracker’s Predicted State.

The motion path can be created between the object’s position state at its current time, to the object’s predicted position at the next time step. This is illustrated in Figure 6. $\hat{x}\left(k|k\right)$ represents the state vector in the kth timestamp. $\hat{x}\left(k+1|k\right)$ is the state prediction at the *k* + 1 time step. 

$\hat{x}\left(k+1|k\right)=\Phi$($\hat{x}$(*k*|*k*)), where Φ represents the motion state matrix.

#### 2.2.3. Motion Path Candidate Evaluation

Once a set of motion paths have been established, we extract a region of pixels (called “chips”) along the path from the Normalized Difference Frames buffer. The size of the chip should be large enough that the number of target pixels are statistically much smaller than the total number of pixels. Ideally, we want the number of target pixels to not exceed 10% of the total number of pixels in the chip. To enhance SNR, we sum up all the chips along the motion path over the framed sequence. Mathematically, this can be expressed as the following:

(6) $$S_{k}\left(i,j\right)=C\left(i+∆i,j+∆j,t-M\right)+\ldots C\left(i,j,t-2\right)+C\left(i+∆i,j+∆j,t-1\right)+C\left(i+∆i,j+∆j,t\right)$$

where S is the summation of the pixel $\left(i,j\right)$ across multiple frames. (∆i, ∆j) corresponds to the shift positions approximated by the motion paths, M represents the frame buffer window for the summation, and k corresponds to the number of motion candidates. 

To find the optimal match of the target’s signal, we use the Z-score [23] to measure the effectiveness of its integration. A Z-score in statistics is a measure of standard deviation above the mean value. 

The mean $\mu_{s}\;$ and standard deviation $\sigma_{s}$ of the sum chip S can be calculated as follows.

(7) $$\mu_{s}=\frac{1}{p}\overset{P}{\underset{p=1}{\sum }}S\left(p\right)$$

(8) $$\sigma_{s}=\sqrt{\frac{1}{P}\overset{P}{\underset{p=1}{\sum }}{\left(S\left(p\right)-\mu_{s}\right)}^{2}}$$

Then, we compute the Z score of the sum chip $Z_{s}$ for each pixel $\left(i,j\right)$ using the following equation:

(9) $$Z_{s}\left(i,j\right)=\frac{S\left(i,j\right)-\mu_{s}}{\sigma_{s}}$$

**Figure 7:**
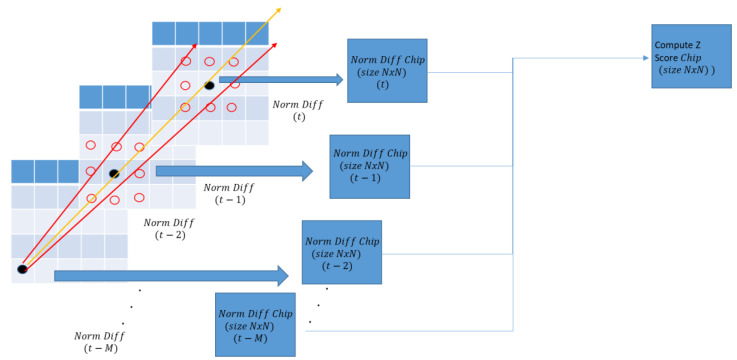
Motion Path Candidate Evaluation.

If the target signal is highly correlated between frames, the integrated result should have a high Z-score. If the integrated result is a poor match, the Z-score would be lower. As each Normalized Difference Frame is integrated, the Z-score of the integrated result will be calculated. If we consistently match the target, additional integrated frames should contribute to an increase in the target’s Z-score. As an illustration, there are three possible candidate motion paths depicted in Figure 7. The “yellow” curve should produce the highest Z-score because the overall target energy spread aligns better over the framed sequence. Finally, we apply a threshold on the sum Z-score chip to detect the target. 

## 3. Results and Discussions

**Figure 8:**
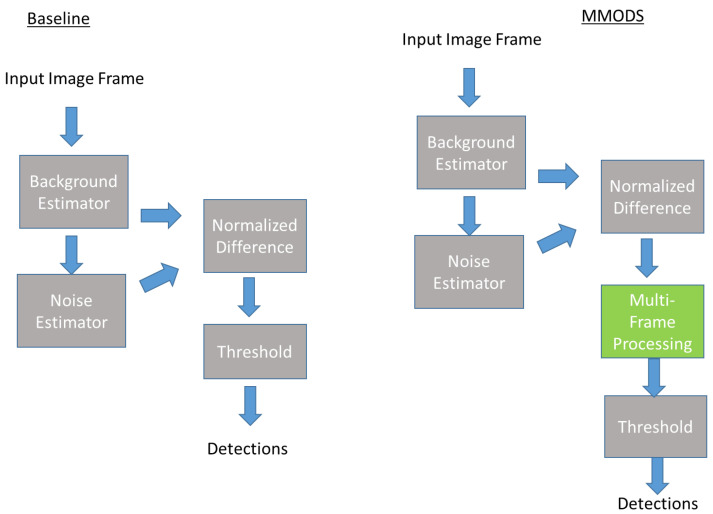
Evaluation Framework: Baseline Architecture vs. MMODS architecture.

To measure the performance improvement of MMODS, we compared a baseline detection system without MMODS versus a detection system with MMODS. A detailed comparison of the system framework is depicted in Figure 8. As depicted in Figure 8, most components were the same except for the MMODS’s design having an additional multi-frame processing component. Both systems used the exact same background estimator in(Equation (1)) and noise estimator in (Equation (2)), and the normalized difference calculation in [Equation (5)]. This comparison setup was used so that we could measure the precise benefit of the MMODS design, as compared to the baseline design. The frame buffer window MMODS used in this experiment was 7.

**Figure 9:**
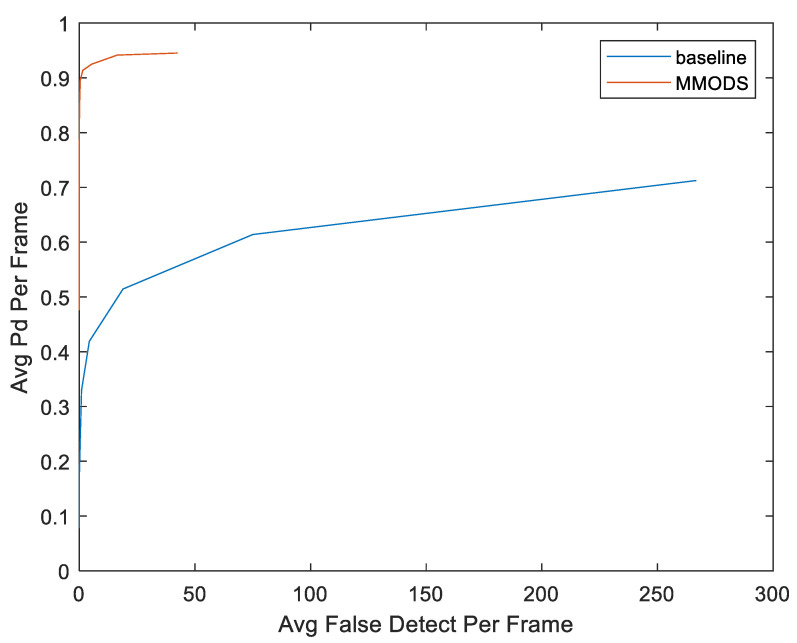
ROC curve comparison using simulated data.

A comparison of the Receiver Operational Characteristic (ROC) curve is depicted in Figure 9. As shown in the curve, to maintain a false alarm of 0, the baseline method could only achieve 30% probability of detection. However, with MMODS, it achieved 90% probability of detection (an improvement factor of 3) at the same false alarm rate. However, we acknowledge that our MMODS method did not reach a probability detection of 100% because some of the targeted SNRs were extremely close, with a 1:1 ratio. 

**Figure 10:**
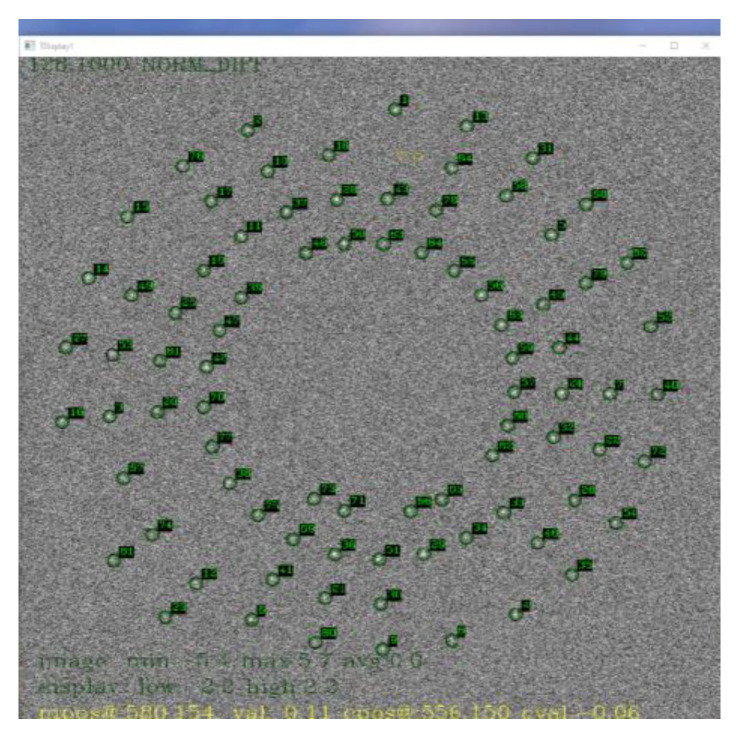
Targets Detected by MMODS overlay on Difference Image.

An overlay of target indicators on the background subtracted image is depicted in Figure 10. The small moving targets were difficult to see on the background subtracted image. 

**Figure 11:**
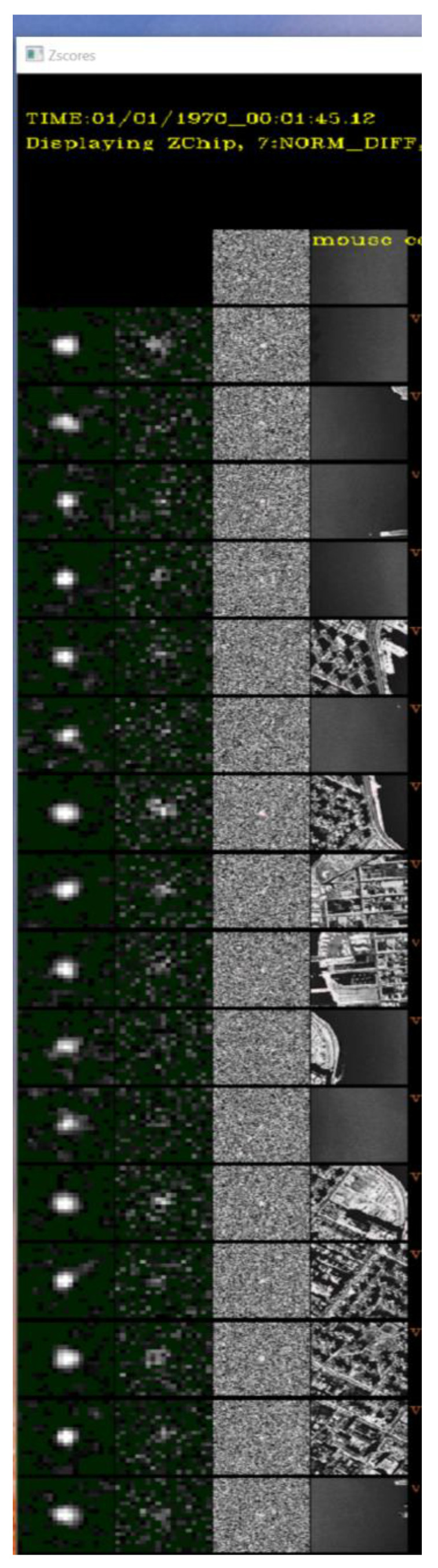
Comparison of Target Chips.

To perform quality assessment, Figure 11 shows a comparison of the MMODS enhanced targeted region (column 1) versus the baseline targeted region (column 2). Both comparison chips were displayed in the same 2× resolution so that we could zoom-in to compare the quality of the SNR enhancement of the small, low-SNR target. When viewed side-by-side, the quality (e.g., SNR) of the MMODS-detected target region in Figure 11, column 1, had a much better quality than the one without MMODS in Figure 11, column 2. For reference comparison, the background subtracted region (column 3) and the original raw frame region (column 4) around the target area is displayed side-by-side in Figure 11 column 3, and Figure 11 column 4, respectively. This again confirms that the targets could not be seen in both background subtracted images and in the original raw image, but it was detected under MMODS.

**Figure 12:**
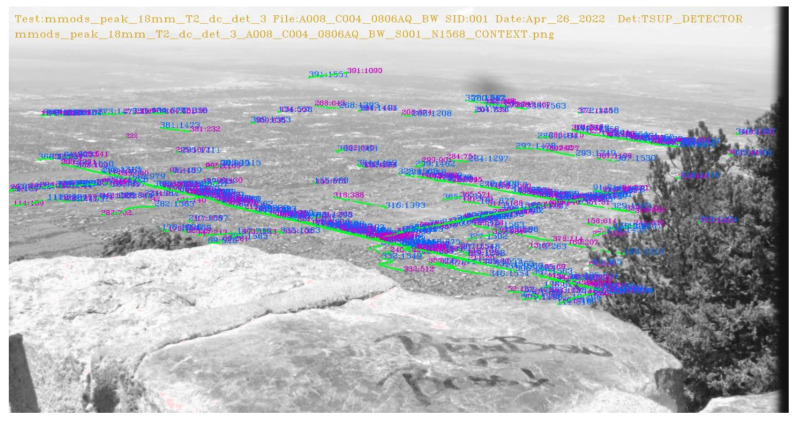
MMODS Detection on Sandia Mountain Peak.

**Figure 13:**
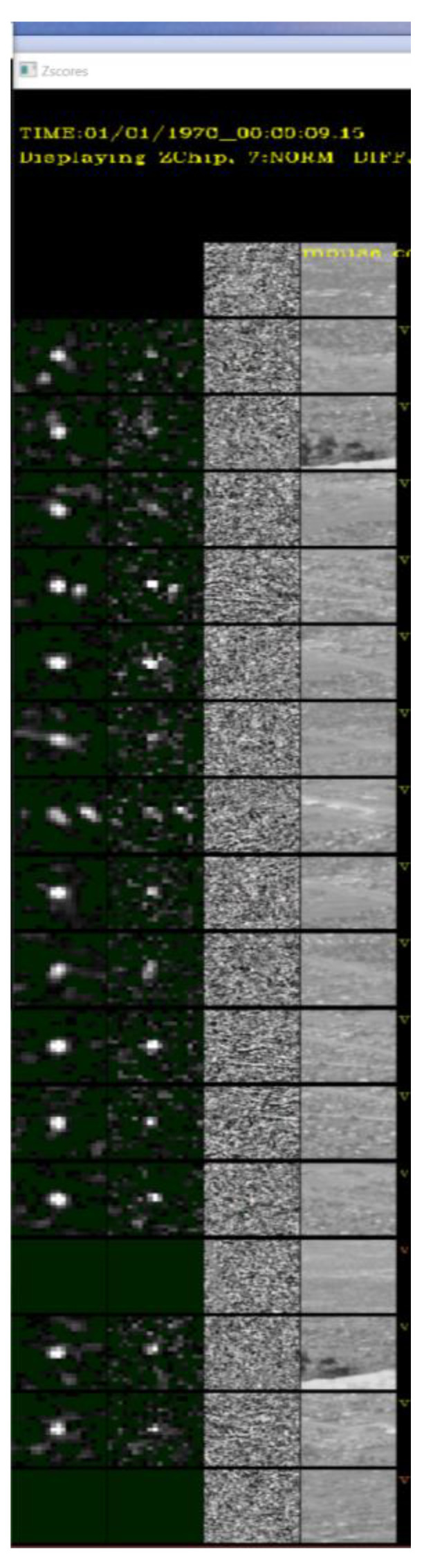
Chip Comparison of Detected Vehicle at Sandia Mountain Peak.

We also processed the video frames we collected at Sandia Mountain Peak in Table 2 using a remote camera with specifications as shown in Table 3 under MMODS. The detections from MMODS across the entire frame history were overlayed on the raw image depicted in Figure 12. With all the detections well aligned with the roads, it is very likely MMODS detects the vehicles because MMODS does not require a priori knowledge about the roads. A comparison of target chips is depicted in Figure 13. The SNR enhancement is very comparable to our simulated results. 

A ROC curve comparison was not generated for this dataset because (1) ground truth vehicle position was not available for this dataset; and (2) we were unable to humanly label the targets with absolute certainty due to poor resolution and low visibility of the targets. However, the fine alignment between the detected position of the targets and the roads, as well as similarities of the detection enhancement in target chips depicted in Figure 13, strongly suggest that MMODS detected true targets with very low false alarm rates on this dataset.

### 3.1. Significance of Contribution and Impact to Modern Remote Sensing System

Detecting small and low visible objects in real time is a challenging task for human-monitored security systems. The task becomes even more difficult if one human analyst must simultaneously monitor multiple displays in real time. Human eyes are not capable of integrating signals to enhance an object’s SNR. If the signal is too low to be recognized by human vision, the target signal might be missed. This innovative technology can intelligently find and correlate signals, and then integrate them across a linear set of video frame sequences to increase the target’s SNR and overcome the LOD. Hence, the MMODS technique can detect signals that cannot be normally observed by the human eye or even by some sensors. 

MMODS was created to help overcome these challenges for remote sensors. MMODS fills in a major gap by providing a new capability to detect the smallest, finest, and lowest visible object that a human would have difficulty identifying in real time. When a sensor is “sensing”, MMODS intelligently matches and integrates target signals as video frames that are flowing into the system to increase an individual target’s SNR. The SNR enhancement is made possible because moving target signals are correlated over a temporal frame sequence, but the noise is generally uncorrelated (random). Hence, when correlated signals are integrated, they increase by a linear factor, but the random noise does not increase by the same amount. By accurately matching and integrating correlated target energy over multiple frames, MMODS overcomes the LOD in a frame-by-frame processing system.

We have demonstrated in a modern PC with a GPU card that MMODS can achieve real-time performance with modern megapixel cameras that detect a large quantity of targets. In comparison, most existing techniques can only support the simultaneous detection of a low number of targets [14,15,16,17,18,19,20]. It is impossible for a human eye to monitor 10 million pixels at a given time, but MMODS can do all this in real time. The MMODS’s distributed computing solution utilizes a GPU which favorably scales large image sizes and many targets. 

We show in Figure 9 that, by using a frame buffer of 7, MMODS can improve modern detection sensitivity over the existing systems by 300%. This technology enhances a sensor’s ability to detect low visibility targets under challenging environmental conditions, such as low-lighting and low intensity conditions, especially in a far range monitoring system where sensitivity generally diminishes. MMODS also provides lower false alarm rates over the current existing system. The frame-by-frame change detection approach [8,9,10,11,12,13] typically employed by sensors is insufficient to detect low SNR targets with a manageable false alarm rate. This is because lowering the detection threshold for each video frame results in high levels of false alarms. MMODS enhances the target’s SNR before applying any detection threshold. This allows the detection system to increase its sensitivity without the same false alarm penalty. Depending on the specific application, both the background estimator and the noise estimator can be replaced with more sophisticated methods to achieve better background and noise modeling. After Difference Frames are normalized by noise, the output can be transmitted to MMODS to further enhance a target’s SNR. MMODS can also act as a SNR booster in a frame-by-frame detection system. 

MMODS can reliably and accurately detect both fast- and slow-moving objects with high accuracy. In comparison, modern theoretical techniques [21,22] are generally limited to slow-moving targets. Furthermore, the modern long exposure configuration used in “Frame-Based Sensors” can enhance the static object’s SNR, but it generally results in a “blur” or “streak” effect [24]. Modern “Event-Based Sensors” can detect fast moving targets by responding to a brightness change in every pixel, but it does not improve a target’s SNR [25]. Hence, a low detection threshold could introduce many false alarms. MMODS, however, improves the target’s detection sensitivity (SNR improvement) without introducing a motion “blur” or “streak” effect, which would probably introduce a large error in a detected object’s position. MMODS can achieve subpixel resolution accuracy in the object’s detected position, which is beyond even the nominal pixel resolution accuracy.

### 3.2. Application to Remote Sensing

MMODS can be realized as a software application that operates on a computer workstation with a Graphics Processing Unit (GPU) card installed. In real-time surveillance monitoring applications, continuous image streams observed by real-time remote sensors can be sent over to the MMODS processing station. The data transfer can be accomplished through various methods, such as transfer through a network cable, transfer data through a network, or downlinking data to a receiver and then to the MMODS. Once the data is received, each image frame can be processed through the MMODS. It can then intelligently match and integrate target signals as video frames flowing into the system to perform SNR enhancement. The detected target location, as well the target’s estimated motion state—such as its predicted position and predicted velocity generated by MMODS—can be outputted to the analyst’s workstation for display and reporting. 

### 3.3. Limitation

While MMODS does not require speed or acceleration to be precisely known, the technology requires a known upper bound in terms of the velocity and acceleration of the targets. This information is usually easily obtainable, such as the maximum speed of a car, plane, etc. The upper bound is used to keep the system from spending wasteful computation searching for an unreasonable target trajectory. If unsure, one can always set a conservative upper bound estimate to help prevent this from happening. There is also a trade-off involved between the number of frames used in the integration window versus real-time latency. While the longer integration window improves SNR, it also increases real-time latency. For example, for a seven-frame integration window [f($t-3),f\left(t-2\right),f\left(t-1\right),f\left(t\right),f\left(t+1\right),f\left(t+2\right),f(t+3$)], where $f\left(t\right)$ represents the frame received at time t, object detection at time t can only be performed after frame $f\left(t+3\right)$ is received, thus contributing to a delay reported by three frames as compared to a frame-by-frame detection system. This additional latency is usually negligible in modern sensors with high frame rates, but it is worth noting in design considerations.

## 4. Conclusions

The MMODS technology fills a major technology gap in remote sensing surveillance applications to enhance the detection of small and low SNR targets. A U.S. patent [26] has been issued for our technology. Our patented MMODS approach combines object detection processing with a dynamic motion estimation algorithm to enhance a target’s SNR. This is accomplished by a smart method to find, match, and integrate target signals over a temporal frame sequence. We have demonstrated through a simulated scenario that our technique provides a factor of 3 improvement in probability of detection over the baseline method. Our method can achieve even greater improvement if we increase the frame buffer beyond the seven-frame integration window we are currently using. In addition, we have demonstrated that our technique can achieve similar improvement in real-world conditions. Our technique does not require pre-labeled data nor prior knowledge about the environment. Our technology only requires minimal a priori knowledge about the objects being sensed, as well as some upper bounds pertaining to the realizable velocity and the acceleration of the targets. This makes our technology very practical for real-time surveillance applications. Moreover, MMODS can be inserted as an additional modularized component that can act as an “SNR booster” into current modern detection systems to further improve probability detection while reducing false alarm rates. For future work, we plan to extend our approach to handle non-linear target movements.

## 5. Patents

The novelty of this work is evident by the recent issuance of a U.S. patent [26]. 

## Figures and Tables

**Table 1:** Simulation Scenarios.

Number of Moving Targets	100
Range of Target SNR Injected	SNR range from 1 to 5
Range of Target Speed	Speed range from 4.0 to 10.0 $\frac{pixel}{sec}$ (at 25 Hz sampling)
Range of Target Acceleration	Acceleration range from 0.086 to 0.217 $\frac{pixel}{sec^{2}}$

**Table 2:** Experiment Location.

Camera Location	Peak of Sandia Mountain, Albuquerque, New Mexico
Camera Location Elevation	10,379 feet
Ground Target Elevation	6060 feet

**Table 3:** Camera Specifications.

Video Camera	Frame Rates	Image Resolution	Lens Focal Length
Mysterium X	24 frames per second	3072 × 1620	18 mm

## Data Availability

Data is currently resided in Sandia National Laboratories. Additional approval may be needed for release of collected data.
